# Design and fabrication of silicon-tessellated structures for monocentric imagers

**DOI:** 10.1038/micronano.2016.19

**Published:** 2016-05-23

**Authors:** Tao Wu, Stephen S. Hamann, Andrew C. Ceballos, Chu-En Chang, Olav Solgaard, Roger T. Howe

**Affiliations:** 1 Department of Electrical Engineering, Stanford University, Stanford, CA 94305, USA

**Keywords:** curved image plane, monocentric imager, photodiode, recessed negative trench profile

## Abstract

Compared with conventional planar optical image sensors, a curved focal plane array can simplify the lens design and improve the field of view. In this paper, we introduce the design and implementation of a segmented, hemispherical, CMOS-compatible silicon image plane for a 10-mm diameter spherical monocentric lens. To conform to the hemispherical focal plane of the lens, we use flexible gores that consist of arrays of spring-connected silicon hexagons. Mechanical functionality is demonstrated by assembling the 20-μm-thick silicon gores into a hemispherical test fixture. We have also fabricated and tested a photodiode array on a silicon-on-insulator substrate for use with the curved imager. Optical testing shows that the fabricated photodiodes achieve good performance; the hemispherical imager enables a compact 160 ° field of view camera with >80% fill factor using a single spherical lens.

## Introduction

### Motivation

Curved image planes that mitigate spherical aberration are essential for wide-angle, monocentric imaging technology to be generally useful^1^. Multiple efforts have been devoted toward developing monocentric imagers. Ford *et al.*^1–3^ designed a monocentric triplet with a spherically polished fiber bundle that conforms to the lens and acts as a spherical-to-planar coupler to a CMOS imager. Crosstalk and the mode volume of the fibers constrain the pixel size and fill factor. These limitations motivate an alternative approach—a curved imaging plane. Sony has developed a curved CMOS imager by controlling bending stress^4^, which shows a 1.4× improvement in sensitivity and a 5× reduction of dark current; however, the radius of the curvature is too large for full hemispherical coverage of a miniature spherical lens. Rogers *et al.*^5–8^ demonstrated a hemispherical camera, consisting of isolated sensor clusters attached to an elastic material; however, the sensor area has a low fill factor, and the pixel density is nonuniform because of stretching. Dinyari *et al.*^9,10^ and Mathieson *et al.*^11,12^ reported a technique to develop curved monolithic silicon focal plane arrays for retinal implants; however, the process requires backside thinning, and metal interconnections have yet to be demonstrated.

In addition to the motivation to achieve a miniature monocentric imager, curved silicon structures can enable a variety of applications in flexible electronics and the Internet of Things^13–15^. Therefore, we seek to address the challenge of developing a low-cost, CMOS-compatible process for silicon-based flexible electronics by demonstrating a miniature hemispherical curved silicon substrate for monocentric imagers.

### Concept

We seek to construct a flexible silicon surface that can conform to a hemispherical shell, by using planar lithographic techniques. Although it is known that a perfect hemispherical shell cannot be constructed by folding a plane, a hemispherical surface can be approximately formed from planar structures by assembling flexible gore segments. The curve for a perfectly conformal gore segment is described by

(1)y=±rtan(π/n)sin(x/r)

where *n* is the number of gores^16^.

**Figure 1:**
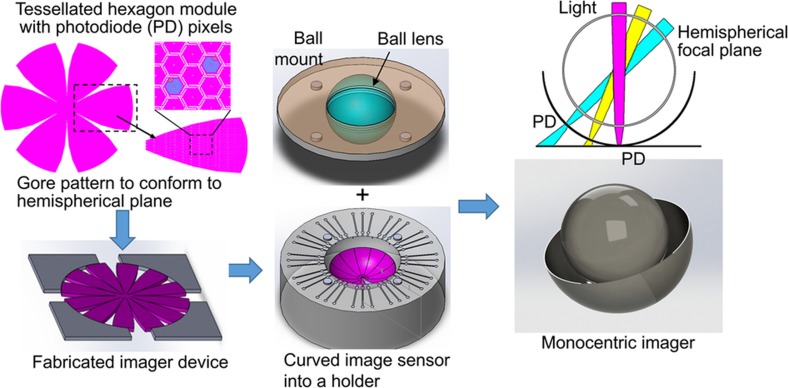
A design concept of a compact monocentric imager, which consists of a tessellated monocentric imager with a customized ball lens and a curved sensor holder. The top-right inset shows a monocentric imager with a hemispherical curved focal plane can simplify the lens design to just a single ball lens.

Figure 1 shows the design concept of a compact monocentric imager. Within each gore pattern governed by Equation (1), tessellated hexagonal structures, connected by spring bridges, form a flexible network. Each hexagonal cell can be up to several hundred microns in diameter. Using current CMOS active-pixel image sensor (APS) technologies with pixel sizes between 1 and 4 μm^17^, thousands of pixels can be fabricated on one hexagonal cell, and hundreds of hexagonal cells can be accommodated in one gore. The bridges function as electrical connections and act as deformable springs that provide mechanical flexibility between semi-rigid hexagonal cells. The top-right sketch of Figure 1 indicates such an aberration-free monocentric imager design with a hemispherical focal plane; the single ball lens can achieve a wide field of view with a significant reduction of the imager size. To demonstrate the manufacturability of this design, we have fabricated and tested photosensitive diodes on the hexagon cells and assembled the tessellated planar silicon into a hemispherical holder with a ball lens.

## Materials and methods

### Structural design and simulation

The use of thin substrates is essential to achieve hemispherically curved silicon surfaces because of the high Young’s modulus and brittle fracture characteristic of silicon. Our fabrication process realizes thin substrates by employing silicon-on-insulator (SOI) substrates, which are compatible with modern CMOS imager technologies^18,19^. The thickness of the device layer is subject to design tradeoffs. A thin device layer leads to better conformity and less stress, whereas a thick device layer provides mechanical strength, mitigates stress concentration and improves optical absorption. A ball lens with a diameter of 10 mm and a refractive index of 1.54 is used for our imager design. The corresponding focal length is ~7.2 mm. Each hexagonal unit cell can vary from tens of microns to hundreds of microns in diameter. The width of the interconnect bridges and trenches must be between 4 and 8 μm to accommodate metal lines of width 2 to 4 μm. Because the hexagonal cell has six directions for interconnection, the design rule is one-half of the spacing and clearance of each layer in conventional designs. This limits the line/space critical dimension in our design and affects the fill factor of the overall image sensor. In one of our primary designs, the diameter of the hexagonal cell is 200 μm, and the line and trench width is 4 μm. The corresponding fill factor is ~80%, assuming that the whole hexagonal unit cell is photosensitive. Optimizing the hexagon cell size and interconnect spring line/space width with improved alignment accuracy can further improve the fill factor.

**Figure 2:**
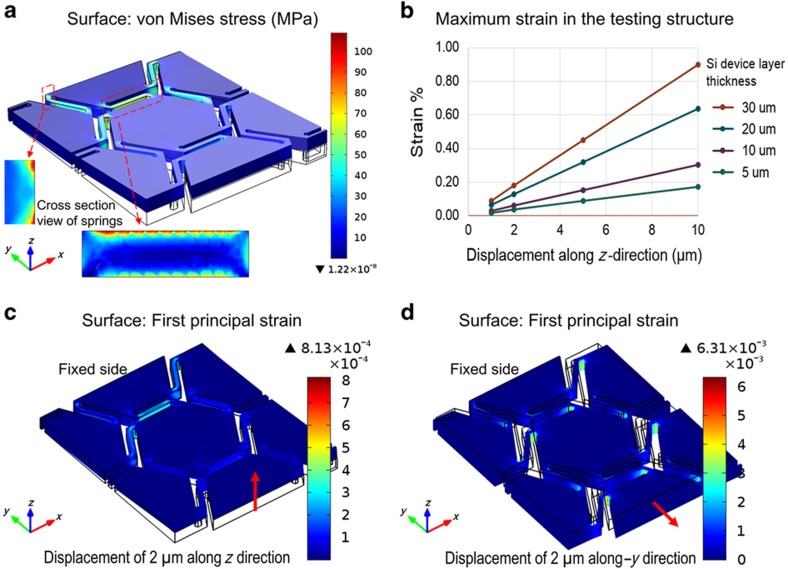
(**a**) Surface stress simulation of a test structure with one end fixed and the other end with a prescribed *z*-direction displacement of 2 μm; two insets show cross-section views of the interconnecting springs’ stress distribution in two different locations; (**b**) the maximum strain concentration of the test structure as a function of *z*-direction displacement with different device layer silicon thicknesses; surface strain distribution with a prescribed displacement of 2 μm; (**c**) along the +*z* direction; and (**d**) along the –*y* direction.

COMSOL Multiphysics^®^ (COMSOL, Inc., Burlington, MA, USA) is utilized to analyze and optimize the stress/strain distribution in the suspension connecting the hexagonal structures. An evaluating test structure of a 20-μm Si device with a complete hexagonal unit cell and six half-cells interconnected by springs represents the tessellated hexagonal structure. Figure 2a shows the surface stress distribution when one side of the structure (half-cell surface in the +*y* direction) is fixed and the other side of the structure has a prescribed displacement along the *z*-direction on the order of 2 μm. This corresponds to the situation in which the planar Si structure is conformed to a hemispherical surface with finite deformation along the *z*-direction on each hexagonal unit cell. The three-dimensional (3D) surface stress distribution plot indicates that stress concentrates on the spring bridges. The main photosensitive area—that is, the hexagonal unit—is mostly stress free. Therefore, the influence of curving stress on photodiodes or potential CMOS transistor circuitry is minimized.

Two enlarged insets in Figure 2a show cross-section two-dimensional views of the springs’ stress distribution in two different locations. A further analysis of the maximum strain versus different displacements along the *z*-direction is presented in Figure 2b. The plot indicates that the maximum strain value of the test structure is linearly proportional to the displacement along the *z*-direction at a given device thickness. The maximum stress/strain can be significantly reduced by using a thinner Si device layer. Figures 2c and d evaluate the strain distribution of the test structure with 2-μm displacement along the +*z* and –*y* directions, respectively. Because our approach does not require stretching of the device to conform to the hemispherical surface, the actual displacement along the *y* direction should be nearly zero. The maximum strain concentration shown in Figure 2d indicates the robustness of the device design subject to external vibration and stretching. With our target 7.2-mm imager plane, the average displacement of each hexagonal unit cell is <2 μm, which results in a maximum strain concentration on the bridge far <1%. Therefore, any device structure thickness <30 μm would be adequate for achieving the required flexibility, with minimal stress concerns.

**Figure 3:**
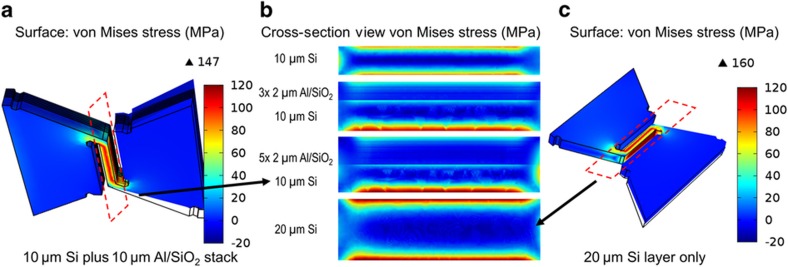
(**a**) Surface stress simulation of a test structure with a 10-μm Si layer and a 5-layer Al/SiO_2_ metal/dielectric stack. Each Al/SiO_2_ layer consists of 0.5 μm of Al and 1.5 μm of SiO_2_. (**b**) Cross-section stress distribution of 4 different stacks: a 10-μm Si layer, a 10-μm Si layer and a 6-μm Al/SiO_2_ stack, a 10-μm Si layer and a 10-μm Al/SiO_2_ stack and a 20-μm Si layer. (**c**) Surface stress simulation of a 20-μm Si test structure.

We further investigate the impact of stress on the metal/dielectric stack in CMOS processes. Although current CMOS backend processes can include 10 or more metal interconnect layers, a common APS technique typically contains three or four metal layers for signal processing and readout^20^. We investigate stress in the metal/dielectric stack by adding multiple Al/SiO_2_ layers atop a 10-μm Si layer, with each Al/SiO_2_ layer consisting of 0.5 μm of Al and 1.5 μm of SiO_2_. As shown in Figure 3, a structure consisting of two half-hexagon cells and an interconnecting spring is simulated. Figure 3a shows the 3D view of the stress distribution in a structure of 10 μm of Si and a 10-μm 5-layer Al/SiO_2_ stack, compared with that in the structure of 20 μm Si in Figure 3c. Similar to the stress simulation in Figure 2a, the maximum stress is present along the sharp spring corner. Along the middle part of the spring, stress concentrates on the surface. Figure 3b illustrates the cross section of the springs on four types of stack structures. It can be seen that stress concentrates along the silicon substrate surface, whereas the interfaces between the metal/dielectric stack have lower stress. This phenomenon results from the lower Young’s moduli of Al and SiO_2_ compared with that of silicon. The total stack thickness also contributes to the maximum stress value. A summary of the maximum stress values of the four types of stack structures in Figure 3b is provided in Table 1. The maximum stress increases with increasing total thickness of the structure, which is consistent with Figure 2b. The minimum thickness of the silicon is constrained by the required depletion depth in the photodiodes. The metal/dielectric layers contribute to the total thickness and increase the maximum stress and strain. Our design uses 20 μm of Si with one metal/dielectric stack as a proof of concept for structural design and process compatibility. From Table 1, the maximum stresses in a 10 μm-thick silicon with a 10 μm-thick, 5-layer metal/dielectric stack, typical of CMOS imagers, are similar to those of our proof of concept design.

### Photodiode design and simulation

**Figure 4:**
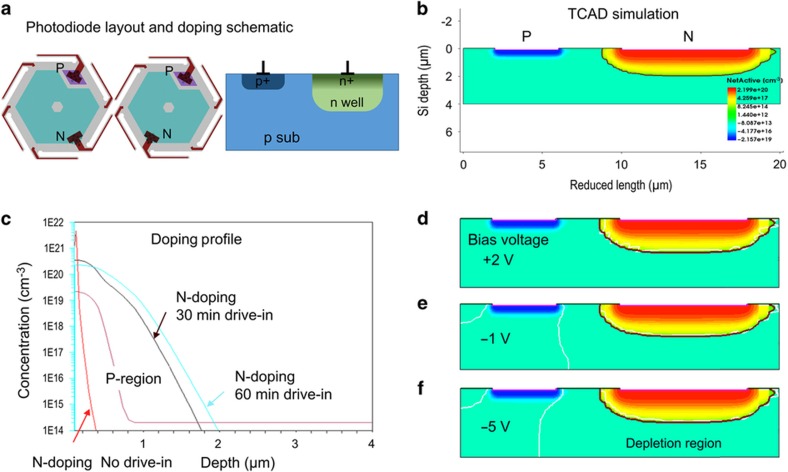
Sentaurus simulation on the photodiode (**a**) layout; (**b**) cross-section doping profile; (**c**) doping profile on the *p*+ and the *n*+ regions after ion implantation and different drive-in conditions; cross-section doping profile and depletion boundary (white line) at a bias voltage of (**d**) +2 V, (**e**) −1 V, and (**f**) −5 V.

To demonstrate that devices made with a CMOS-compatible process could function properly and maintain performance with the flexure of the substrate, we fabricated and tested basic photodiodes on an SOI substrate. The process employs a modified *p* substrate and *n* well design. Pinned photodiodes, which are used in commercial CMOS imagers^21^, are compatible with this process and can be included in future device implementation. Various diode layouts were evaluated using the Synopsys^®^ Sentaurus TCAD simulator (Synopsys Inc., Mountain View, CA, USA) to optimize the process design. Figure 4a shows two photodiode layouts and a schematic cross-section doping profile. Figure 4b shows the TCAD simulation results on the depletion region. The dimensions used in the simulation are scaled except for the PN junction spacing to reduce computation time. Figure 4c shows the doping profile of the *p* and *n* regions. The simulated junction depth after 60 min of annealing is ~1.9 μm. The *n*+ region is formed in a gas-phase doping furnace using POCL_3_ as a phosphorus source, and the surface phosphorus concentration is as high as 1×10^20^ cm^−3^ to make ohmic contact with the subsequent metal layer. A heavily doped p+ region is formed by boron ion implantation at 20 keV with a dose of 5×10^15^ cm^−2^. A surface boron concentration of 2×10^19^ cm^−3^ is achieved. Figures 4d–f show the simulated depletion region of the PN junction with a bias of +2, −1, and −5 V, respectively. As the reverse bias voltage increases, the depletion region widens and moves closer to the *p*+ region. The junction depth extends several microns deep at −5 V, yielding sufficient photoabsorption for the proof of concept device.

### Photodiode addressing

**Figure 5:**
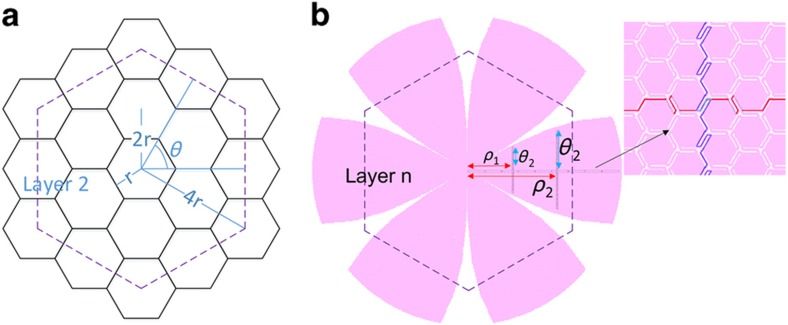
(**a**) The tessellated hexagon network with layered addressing concept; (**b**) a six-gore device design using a polar-like coordinate system (*ρ*,*θ*) to address individual hexagonal cells; the inset shows enlarged two-layer metal routing along the interconnect sprints.

Because of its unique hexagonal geometry, conventional row-column addressing is inappropriate for this imager. Inspired by the flower-shaped gore patterns described by Equation (1), we propose a polar coordinate system to address the individual hexagonal unit cells of the curved imager. As shown in Figure 5a, for a fully covered tessellated hexagonal network with a hexagonal unit cell of edge radius *r*, the hexagon center is the origin and is defined as layer 0, a discrete radius *ρ* represents different layers along the polar axis, and the angular position *θ* is determined by the numbering of the cell times the cell angle at each layer, as listed in Table 2. Therefore, the radius *ρ* and angular angle *θ* coordination for any hexagon cell at layer *n* can be expressed at (2*nr*, 2π*m*/6*n*), where *m* is an integer <6*n*.

Two additional factors remain. First, as mentioned in the previous section, we fabricated one photodiode on each single hexagonal unit cell as a proof of concept, and the diameter of each hexagonal unit cell ranges from 100 to 200 μm, which is chosen by balancing the fill factor and release etching time. However, the pixel sizes of modern CMOS imagers have decreased to ~1 μm or less, so each hexagonal unit cell could contain thousands of modern pixels, arranged in a continuous, planar silicon substrate. Thus, each hexagon cell can be treated as a group of thousands of pixels laid out on a row-column grid.

Second, the gore pattern has its own boundary governed by Equation (1). As the radius increases, the number of hexagonal cells will decrease to less than the full-coverage quantity 6*n*. The tessellated network is divided into several sections according to the numbers of gore patterns. Thus, each gore pattern could require one signal bus along the radial axis to address different layers.

Figure 5b shows the polar addressing coordinate for the hexagonal cells. In the first gore pattern, a bus line connects the origin and the outer edge through the interconnected spring hinges and hexagonal cell edge. The distance is defined by the polar length *ρ.* At different polar positions, another signal line is extended into each individual cell with a unique angular coordinate *θ*. Only two metal layers are needed to connect all hexagonal cells.

There are several strategies to achieve seamless coverage on a spherical surface. One approach is to design gore patterns governed by Equation (1) that perfectly align each other and conform to a hemispherical surface. This approach requires very precise assembly. Another approach is to use two patterns of *n*/2 center-connected segments that are slightly wider than Equation (1), and two patterns can overlap below on one side and above on the other. The addressing bus lines and readout circuitry could be laid out in this area without sacrificing usable photosensitive area.

### Fabrication

**Figure 6:**
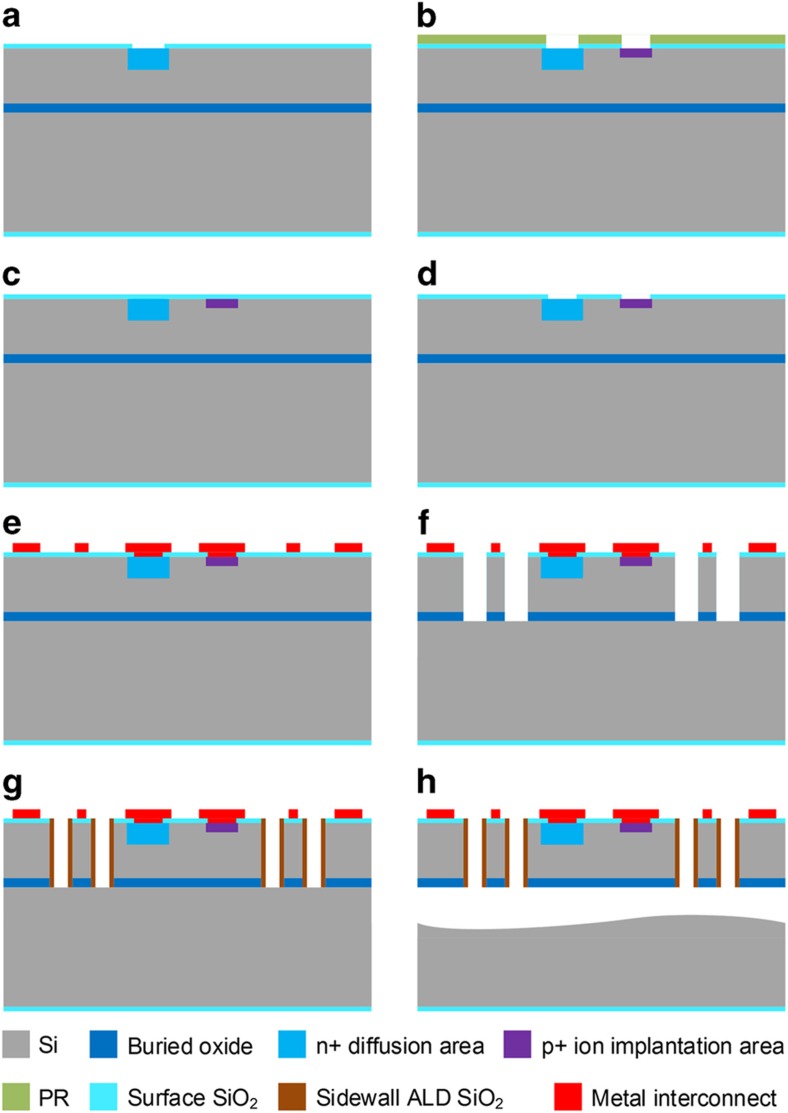
Fabrication process flow: (**a**) form n+ diffusion area; (**b**) form p+ ion implantation area; (**c**) grow new surface oxide; (**d**) pattern via for photodiode contacts; (**e**) deposit metal contact and interconnect; (**f**) pattern tessellated structures thru Si device and buried oxide layers; (**g**) sidewall passivation; (**h**) release the device by XeF_2_ etching.

The fabrication process starts on SOI wafers with a 20-μm-thick device layer and a 0.5-μm-thick buried oxide (BOX). The device layer resistivity is 50–100 Ωcm. The process flow is shown in Figure 6 (see Supplementary Information for process equipment details and recipe conditions). First, 100-nm of thermal SiO_2_ is grown and patterned as a mask for n+ doping. A phosphorus predeposition process is performed with a Tylan POCl3-doping furnace followed by an annealing step at 1000 °C for 30 min, resulting in a sheet resistance of ~5 Ω sq^−1^ on the n+ phosphorus doping region (Figure 6a). This forms a PN junction and also provides a good contact for the n+ region to the metal layer. Another thermal oxidation step is performed and patterned to define the opening for p+ boron implantation (Figure 6b). The ion implantation is conducted using B^11^ singly charged boron at 20 kV at a dose of 5×10^15^ cm^−2^. After ion implantation, a new 100-nm surface thermal oxide is grown (Figure 6c), followed by a 20-min drive-in step at 1000 °C, resulting in a sheet resistance of approximately 5 Ω sq^−1^ on the p+ phosphorus-doping region. Vias are then opened by etching through surface oxide layers from the contact regions (Figure 6d). Following that, a metal layer of 10-nm Ti/100-nm Pt is deposited and patterned by a liftoff process to form the interconnect (Figure 6e).

After the photodiode circuitry has been fabricated, ~250-nm of PECVD SiO_2_ and 50-nm of ALD Al_2_O_3_ are deposited atop the device as a surface dielectric stack and etch buffer layer. The tessellated hexagonal structure is patterned using a 3-μm photoresist as a mask followed by reactive ion etching (RIE) on surface Al_2_O_3_ and SiO_2_ stack layers to expose the device silicon layer. The silicon layer is etched by deep RIE (DRIE) followed by another RIE etching of the 0.5-μm barrier oxide layer (Figure 6f). CMOS imagers include a thick metal-dielectric stack, which will require switching plasma etch chemistries during the hexagonal structure etch^22^. The thickness of the surface oxide etch buffer layer can be increased to allow for deeper etch depth. Alternatively, a temporary metal hard mask can be added.

Following the structure definition, a conformal coating of a pinhole-free, 50-nm ALD Al_2_O_3_ layer, and a 250-nm PECVD SiO_2_ or low-temperature oxide (LTO) layer are deposited as sidewall passivation and protection layers for the release etch. A directional RIE etch step is performed to remove SiO_2_ and then Al_2_O_3_, exposing the silicon handle substrate at the bottom of the trenches and giving access to the XeF_2_ release etch (Figure 6g). The structure is released by XeF_2_ dry etching (Figure 6h). Finally, a conformal coating of 5–10 μm Parylene-C film is deposited to improve the mechanical robustness of the gores, allowing them to be transferred and mounted into a hemispherical device holder.

**Figure 7:**
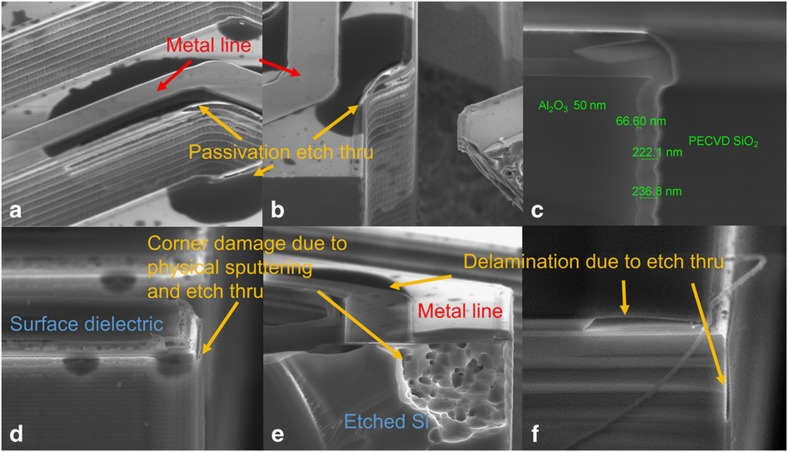
SEM images of devices showing (**a** and **b**) surface dielectric and sidewall passivation delamination; (**c**) ultrasmooth sidewall passivation with 50-nm ALD SiO_2_ and 250-nm PECVD SiO_2_; (**d**–**f**) trench corner damage due to enhanced etching.

A high quality and CMOS-compatible sidewall passivation process is critical for the device release step. Our process uses ALD Al_2_O_3_ and PECVD SiO_2_ or LTO conformal films. ALD Al_2_O_3_ has better adhesion to silicon surfaces than other ALD films, but the ALD layer thickness is limited because of its very low deposition rate. A second low-stress layer of PECVD SiO_2_ or LTO is used to improve the sidewall passivation integrity and provide protection for the ALD Al_2_O_3_ etch. The etching species for Al_2_O_3_ are Cl_2_ and BCl_3_, which etch many other materials. Significant delamination was observed initially because of poor sidewall passivation, as shown in Figures 7a and b. This problem was attributed to a relatively large scallop depth arising from the standard DRIE etch process. To mitigate delamination, we improved the silicon sidewall smoothness. A post-etch hydrogen annealing process requires very high-temperature annealing, much too high for CMOS compatibility. Therefore, we optimized the sidewall profile by utilizing the fast switching feature in a PlasmaTherm Versaline DSE silicon DRIE etcher. Figure 7c shows that a sub-100-nm scallop depth can be achieved with a spacer stack of 50-nm of ALD Al_2_O_3_ and 250-nm of PECVD SiO_2_.

However, even with a smooth trench sidewall profile, a number of defects are seen around the trench corners because of poor dielectric coverage over the metal lines and an enhanced etch rate on sharp edges, as indicated in Figures 7d–f. However, gas transport to the bottom of the trench slows the etch rate and results in incomplete etching. To overcome these challenges, we designed a recessed negative trench profile that successfully protected the ALD Al_2_O_3_ and PECVD SiO_2_ or LTO films near the top corner.

**Figure 8:**
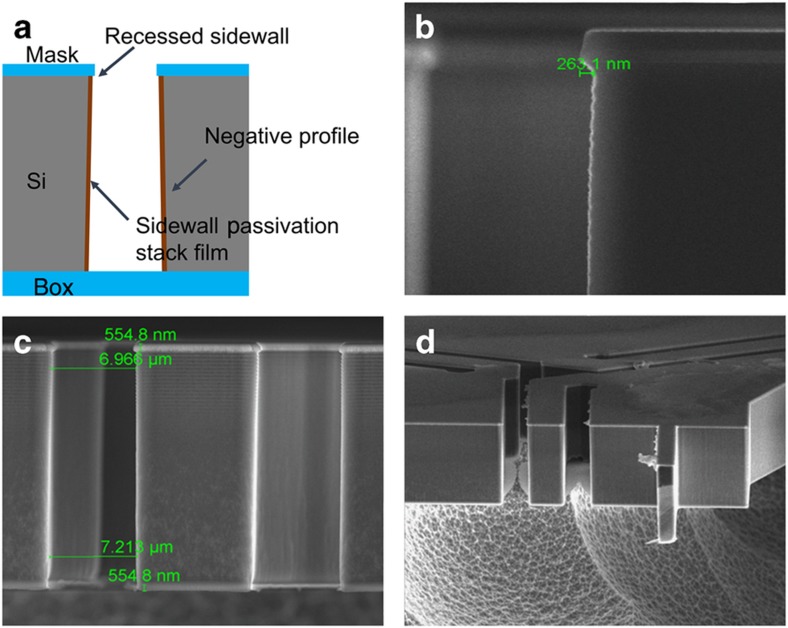
(**a**) The concept of recessed negative trench profile to preserve spacer film and quality; (**b**) an SEM image shows a recessed sidewall profile before passivation coating; (**c**) an SEM image of patterned SOI device layer with a recessed negative profile and passivation coating; (**d**) an SEM image of a fully released structure after XeF_2_ etching.

Figure 8a shows the sketch of a recessed negative sidewall profile. The sidewall passivation stack films, ALD Al_2_O_3_ and PECVD SiO_2_ or LTO, are coated underneath the mask with a recession of 200–300-nm, which matches or is slightly larger than the stack-film thickness. This approach protects the passivation stack films from the physical sputtering near the corner edge. This sidewall recession depth can be tuned by timed etching with CF_4_-enriched gases after DRIE patterning. Figure 8b shows a sidewall recess of ~260-nm achieved using SiO_2_ as the top etching mask. Owing to the difficulty of achieving a perfect vertical sidewall profile, a slightly negative profile is achieved by linearly increasing the etching cycle time of DRIE in the PlasmaTherm DSE tool. Figure 8c shows the zoomed-out image of the trench profile with an ~2 ° negative sidewall, which has a negligible impact on the structural strength and functionality. Figure 8d shows a cross-section image of the released structures after XeF_2_ etching.

## Results and discussion

**Figure 9:**
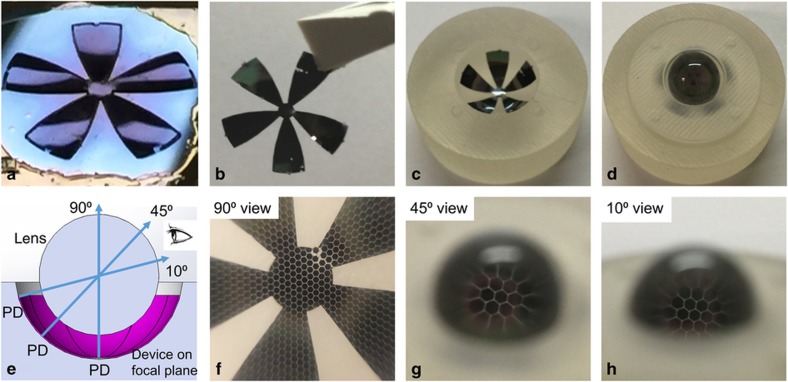
(**a**) A released five-gore structure, still attached to the silicon substrate with tethers; (**b**) a fully released five-gore structure before mounting into the fixture; (**c**) a released and curved device transferred into a hemispherical fixture; (**d**) a mounted monocentric imager; (**e**) a sketch illustrating the ray tracing approach by viewing through a ball lens into released structure; and images taken by a commercial camera through the ball lens at an angle of (**f**) 90 ° (**g**) 45 ° (**h**) 10 ° with respect to the horizon.

The released tessellated structure has tether supports connected to the substrate, as shown in Figure 9a. By carefully breaking those tethers, the 20-μm-thick silicon device with tessellated hexagonal structures is transferred and mounted in a 3D printed plastic test fixture, as shown in Figures 9b–d. The fixture is manufactured using a high-resolution 3D printer (ProJet^®^ 3500 HD, 3D Systems Corporation, Rock Hill, SC, USA). Instead of reconstructing a photodetector mapping, the monocentric imager concept and performance can be preliminarily evaluated through a simple ray-tracing approach: the hexagonal structures on the hemispherical surface are imaged through the ball lens, as shown in Figure 9e. Figures 9f–h are images taken by a commercial camera at viewing angles of approximately 90 °, 45 °, and 10 °, respectively. All three images show clearly visible hexagonal structures, verifying that the released gore is located on the hemispherical image plane. Using this technique, we verified that the field of view is ~160 °. Notably, the focal length can be adjusted by choosing a different ball lens material and refractive index.

Figure 9f also indicates that fracture could occur during the transfer and assembly process, especially at the corner edges near the gore patterns. To minimize the possibility of damage during the assembly of released gores, the fixture’s hemispherical surface is designed with a slightly smaller curvature. A half-hemispherical coverage is more preferable, in which two half-coverage sets of gores overlap each other to achieve full hemispherical coverage.

**Figure 10:**
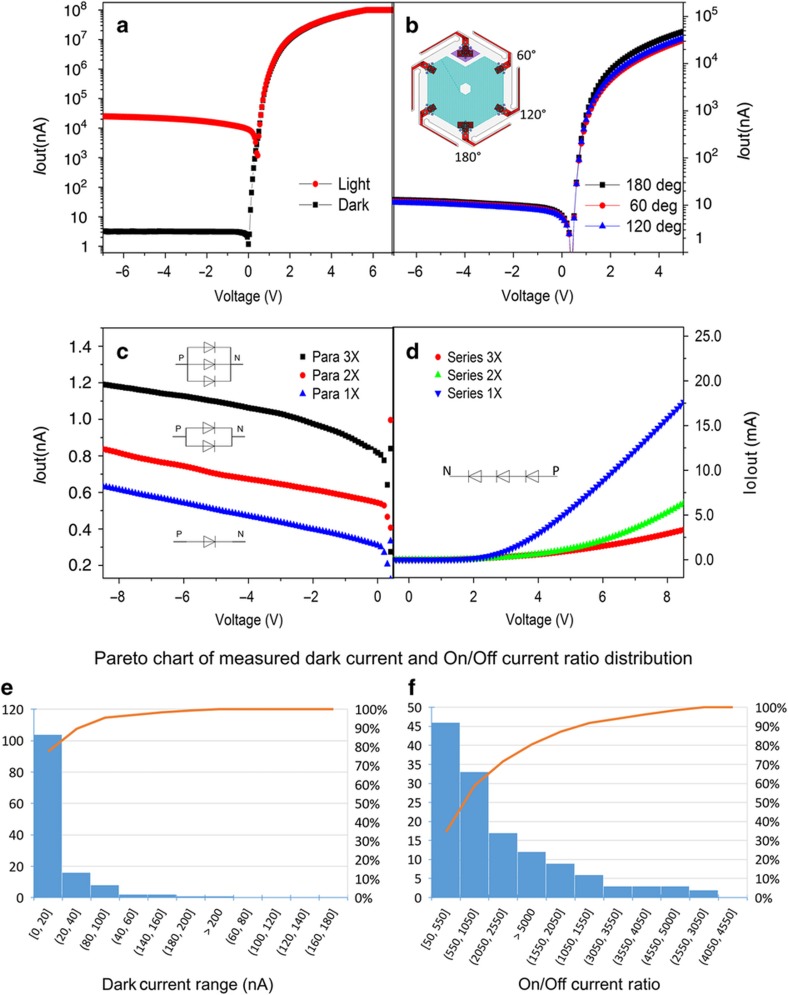
*I*–*V* characteristics of (**a**) one photodiode under microscope light and background dark environment, and (**b**) one photodiode on current under microscope light probed at a fixed p-region contact and different n-region contacts; *I*–*V* characteristics of one, two, and three photodiodes in (**c**) parallel connection and (**d**) series connection; Pareto chart of measured (**e**) dark current and (**f**) on/off ratio distribution.

The optical performance of the fabricated photodiodes is characterized by measuring the current-voltage (*I*–*V*) characteristics in the dark and under illumination. Most of our measurements were taken using illumination under a microscope and were performed before the release etch. Selected photodiodes were wire bonded for characterization after release. Figure 10a shows a typical characteristic of one photodiode in the dark and with microscope illumination. The dark current without background light is approximately 2–3 nA. A reverse bias voltage as high as 20 V is observed without breakdown. With only moderate microscope light illumination, the measured photocurrent is approximately 20–30 μA, resulting in an on/off ratio of approximately 1000:1.

Figure 10b shows *I*–*V* curves of one photodiode from different n-region contacts. The *I*–*V* curves are similar to each other, indicating that the surface potential is uniform across the n-doped diffusion area. This provides the flexibility to access the n-side contact from any of the hexagon cell springs that could be used for the routing network. Figures 10c and d plot the *I*–*V* curves for up to three diodes in parallel and series, respectively. The parallel configuration (Figure 10c) clearly shows that the photocurrent at a negative bias voltage is tripled or doubled at 3× or 2× parallel connection, respectively. The series configuration (Figure 10d) indicates that the series resistance increases with multiple diodes connected in series and increasing diode operating forward voltage. These *I*–*V* characteristics show that good photodiode circuitry can be fabricated in our CMOS-compatible process and conformed to hemispherical surfaces for monocentric imager applications.

Figures 10e and f show the distribution of measured photodiodes before release. The majority of tested devices show a dark current <20 nA, whereas the on/off ratio has a larger range, with the majority falling between 100 and 1000. This discrepancy could be due to several factors, such as process deviation, sidewall passivation uniformity and metal contact resistance variations.

**Figure 11:**
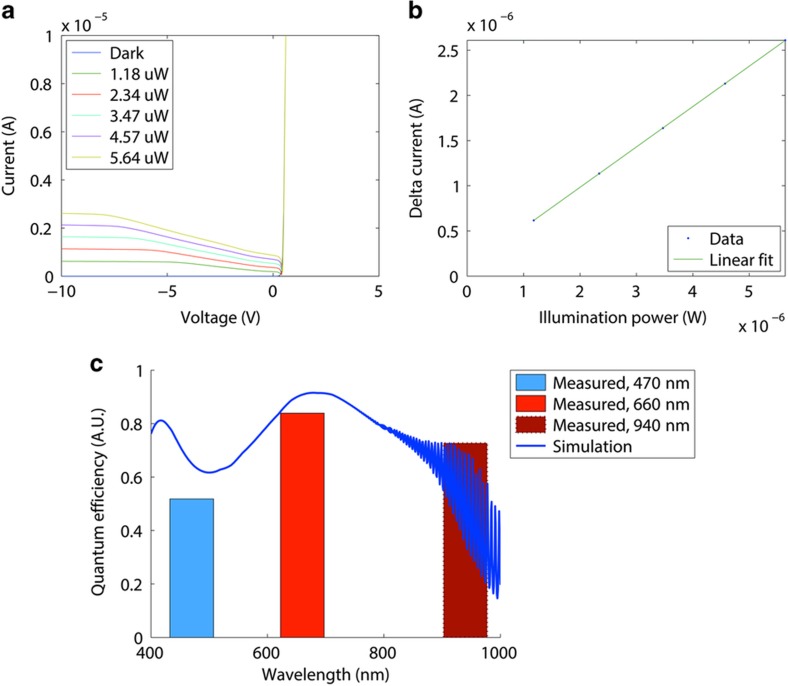
(**a**) *I*–*V* characteristics of a single hexagon cell under 660-nm LED light. The light source is a Thorlabs M660F1. The optical power, at the end of the microscope objective, is measured by a Newport 818-ST2 photodiode with a Newport 1835-C power meter. The ambient light is below 0.1 nW cm^−2^. The displayed power readings have been adjusted to account for the size difference between the photosensitive area of the device and the spot size of the beam; (**b**) Current at −10 V versus incident power, with dark current removed. The response is linear with a correlation coefficient >0.99999, suggesting linear minority carrier generation and collection; (**c**) Measured quantum efficiencies calculated from measured responsivities, compared with simulated results from a 1D transfer matrix method. The simulated structure is, from top to bottom, 350-nm of SiO_2_, 20-μm of Si, 500-nm of SiO_2_, and 550-μm of Si. The structure corresponds to the nominal layer thicknesses described in the fabrication section. Published optical constants^23^ are used in the calculation. Light sources used in the measurement are Thorlabs M470F1, M660F1, and M940F1. 1D, one-dimensional.

Figures 11a and b show a set of *I*–*V* measurements under calibrated illumination, demonstrating the linearity of the photodiode. A 660-nm light-emitting diode (LED) is coupled through a multimode fiber and a microscope objective to the device. The total incident power is estimated by scaling the measured LED power with the ratio of the device’s photosensitive area to the test beam’s spot size, assuming a uniform intensity profile. The gradual saturation of the reverse current is characteristic of the photodiode’s geometry. As depicted in Figure 4, the depletion region expands vertically and improves photoelectron collection with increasing reverse bias. As shown in Figure 11b, with sufficient reverse bias, saturated reverse current is linear to incident illumination. The saturation behavior can be optimized by introducing modern pinned photodiodes and substrate doping engineering into our process. The slope in Figure 11b—namely, the responsivity—allows the quantum efficiency to be estimated. Figure 11c compares the quantum efficiencies at 470, 660, and 940-nm, each measured with a different light source, with the simulation results from a one-dimensional transfer matrix method implemented in-house. The simulated curve shows interference effects that are characteristic of our basic stack structure. The measured quantum efficiencies approximately match the simulation, indicating potential for further optical optimization.

## Conclusion

We have demonstrated a fabrication process for a tessellated gore structure with interconnected hexagonal cells capable of conforming to the hemispherical image plane of a miniature ball lens. In addition, we have shown the imaging capability of this device. Optical testing shows that the fabrication process that allows hemispherical coverage does not compromise the ability to form functional photodetectors. It remains to develop a process for fabricating a hemispherical CMOS imager based on arrays of interconnected hexagonal cells.

## Figures and Tables

**Table 1:** Maximum stress values in the spring

Stress value (MPa)	10-μm Si layer	10-μm Si, 3×2 μm Al/SiO_2_ stack	10-μm Si, 5×2-μm Al/SiO_2_ stack	20-μm Si layer
Max stress inside the spring	117	138	147	160
Max stress in the cross section view of the spring	95.8	99.4	109	121

**Table 2:** Summary of radius and cell angle for each tessellated hexagon network layer

Layer	Radius *ρ*	Number of cells	Cell angle *θ*
Layer 0	0	1	NA
Layer 1	2*r*	6	2π/6
Layer 2	4*r*	12	2π/12
Layer *n*	2n*r*	6*n*	2π/6*n*
