# Supplementary material for: Design and fabrication of silicon-tessellated structures for monocentric imagers
Source: Microsyst Nanoeng. 2016 May 23;2:16019. doi: 10.1038/micronano.2016.19 (PMC6444745; doi:10.1038/micronano.2016.19)
Supplement: Supplementary Information 1 [file micronano201619-s1.pdf]

Supplementary file

Design and fabrication of silicon-tessellated structures for monocentric imagers

Tao Wu, Stephen S. Hamann, Andrew C. Ceballos, Chu-En Chang, Olav Solgaard and Roger T. Howe

Microsystems & Nanoengineering (2016) **2,** 16019; doi:10.1038/micronano.2016.19

Detailed fabrication process flow:

-See a separate Gantt chart file: Monocentric_Gantt_Chart_2015.pdf

All process steps are conducted in Stanford Nanofabrication Facility (SNF) except an ion implantation step outsourced to a vendor INNOViON Corporation

List and detail of equipments can be found in https://snf.stanford.edu/SNF/equipment

Tool and Recipe conditions:

Tool	Purpose	Recipe	Condition	Notes or Depo/Etch rate(ER)
Drytek2	Photoresist descum	Descum	O2: 100 sccm<br>Pressure: 150 mT<br>RF power ~ 83 W	Std recipe
amtetcher	SiO2 etch	Prog3	O2/CHF3: 6/85 sccm<br>Pressure: 40 mT<br>Bias: -530 V<br>RF power: 1600 W	ER ~ 35 nm min^{-1}
Memorase C-91LD	Photoresist curing under 254 nm UV	NA	254 nm UV<br>Exposure 5 ~ 10 min	Harden the photoresist
CCP	PECVD SiO2	SIO350-1	Pressure 1100 mT<br>Power 200 W<br>5% SiH4 flow: 250 sccm<br>N2O flow: 1700 sccm<br>Temp: 350 °C	~ 75 nm min^{-1}
savannah	ALD Al2O3	Al2O3 standard	TMA pulse 0.015 s<br>Wait 20 s<br>H2O pulse 0.015 s<br>Wait 20 s<br>Temp: 200 °C	~ 1 Å/cycle
tylan6	Phos Predep (doping)	POCL900	Temp = 900 °C<br>N2 = 3.7 SLM<br>POCL3 default rate	To form n+ doping Standard recipe only change time
thermco1/2	Oxidation	1/2wetox	O2HI = 7 SLM<br>H2 = 3 SLM<br>Temp = 1000 °C	Standard recipe only change temp and time
tylan9	Forming gas anneal	FGA300	N2: 3.0 SLM<br>H2/N2 = 0.5<br>Temp = 300 °C	Std recipe<br>Change time only
Ion implantation	To form p+ doping		20 keV B(11)+, dose 5e15 cm−2	Outsource to INNOViON Corporation (San Jose, CA, USA)
PT-OX	SiO2 etch	TW_Ox1	O2/CHF3 2/45 sccm<br>Pressure: 5 mT<br>ICP Power: 600 W<br>Bias: 50 W	Oxide ER: ~ 240 nm min^{-1}
	Residue and Si etch	TW_Nit	O2/CF4 5/50 sccm<br>Pressure: 15 mT<br>ICP Power: 800 W<br>Bias: 100 W	Clean up most polymer residue and junk at the bottom of the trench
PT-MTL	Al2O3 etch	TAO_Al2O3	BCl3: 15 sccm<br>Pressure: 3 mT<br>ICP power: 600 W<br>Bias: 100 W	Al2O3 ER: 50 ~ 60 nm min^{-1}

Department of Electrical Engineering, Stanford University, Stanford, CA 94305, USA.
Correspondence: Roger T. Howe (rthowe@standford.edu)

PT-DSE	Si DRIE	Nano_TW	Bosch process loop: Dep/EtA/EtB time: 1/1.5/1.5 s with morph feature ramping to 1/2/2 s<br>Dep/EtA/EtB SF6 flow: 150/150/50 sccm<br>Dep/EtA/EtB C4F8 flow: 150/150/150 sccm<br>Dep/EtA/EtB Pressure: 30/35/25 mTorr<br>RF bias voltage: 10/200/10 V<br>ICP Power: 1500 W	A pre etch step EtB of 1.5 s to control the undercut<br>Negative trench profile is achieved using morph feature to linearly increase the etch time of EtA/EtB steps inside loop<br>Total 90 ~ 100 cycles to etch 20 μm silicon
xactix	Si etch using XeF2	Std recipe	XeF2 Pressure: 3 Torr<br>Etch time per cycle 30 s	120 ~ 150 cycles to release
