# Supplementary material for: Design and fabrication of silicon-tessellated structures for monocentric imagers
Source: Microsyst Nanoeng. 2016 May 23;2:16019. doi: 10.1038/micronano.2016.19 (PMC6444745; doi:10.1038/micronano.2016.19)
Supplement: Supplementary Information 2 [file micronano201619-s2.pdf]

Project Planner

Description	Parameter	Tool
Start from SOI 20um/0.5um/500um; very lightly p-doped resistivity 50~100 ohm.cm		
Pre-oxidation clean	standard (std) RCA clean process	wbclean1/2
Oxidation	1/2wetox, 1000°C 10 mins	thermco1/2
	**Litho N-region**	
Vapor prime	150°C 35 mins	YES oven
Lithography	std process 1 µm 3612 + 1.1 s exposure + dev	svg/KS
Descum	recipe DESCUM 30 sec	Drytek2
Oxide etch	Prog 3, 100 nm oxide etch ~3 mins	amtetcher
PR removal	gasonics std recipe 013	gasonics
Piranha clean	H2SO4:H2O2 9:1 120°C 10 mins	wbnometal
Pre-diffusion clean	standard RCA clean process	wbclean1/2
Phosphorus predepo	Recipe: POCL900 for 30 mins	Tylan6
Annealing	1000°C 30 mins	thermco1/2
Oxide strip	BOE 6:1, ~70 sec (~90 nm/min)	wbnometal
Pre-diffusion clean	standard RCA clean process	wbclean1/2
Oxidation	1/2wetox, 1000°C 10 mins	thermco1/2
	**Litho P-region**	
Vapor prime	150°C 35 mins	YES oven
Lithography	std process 1 µm 3612 + 1.1s exposure + dev	svg/KS
Descum	recipe DESCUM 30 sec	Drytek2
Oxide etch	Prog 3, 100 nm oxide etch 3 mins	amtetcher
Ion Implantation	20 keV B11 dose 5e15 cm-2 (outsource)	INNOViON Corporation
PR removal	gasonics 013	gasonics
Piranha	H2SO4:H2O2 9:1 120°C 10 mins	wbnometal
Oxide strip	BOE 6:1, ~70 sec (~90 nm/min)	wbnometal
Pre-diffusion clean	standard RCA clean process	wbclean1/2
Oxidation	1/2wetox, 1000°C 10 mins	thermco1/2
Annealing	1000°C for 20 mins	thermco1/2
	**Litho Via**	
Lithography	std process 1 µm 3612 + 1.1s exposure + dev	svg/KS
Oxide etch	100nm Via oxide etch by amtetcher or P5000	amtetcher/P5000
PR removal	gasonics 013	gasonics
Clean	Piranha clean and SRD	wbnometal
	**Litho Metal**	
Vapor prime	150°C 35 mins	YES oven
LOL spin coat	LOL2000 1500 rpm	Headway
Bake	Oven 200°C 30 mins	Oven
Lithography	std process 1.6 µm 3612 + 1.6s exposure + dev	svg/KS
Descum	recipe DESCUM 30 sec	Drytek2
Metal deposition	evaporate 10 nm Ti / 100 nm Pt	IntlVac
Lift off	Acetone clean and then soak in 1165(or Remover PG) at 80°C	wbsolvent
Clean	IPA and DI water clean, SRD	SRD
Annealing	Forming gas annealing	tylan9
	**Litho Active**	
Surface oxide	deposit ~250 nm PECVD SiO2 5 mins	ccp
Surface passivation	deposit ~50 nm ALD Al2O3 500 cycles	Savannah
Vapor prime	150°C 35 mins	YES oven
Lithography	std SPR220 3 µm + 3 sec expoure + dev	svg/KS
UV curing	UV curing 5 mins	Memorase® C-91LD
Al2O3 etch	50 nm ALD Al2O3 etch	PT-MTL
Oxide etch	~350 nm surface oxide etch CHF3/O2 based gas	PT-OX
DRIE	20 µm Si etch with morph feature	PT-DSE
Oxide etch	500 nm barrier oxide etch CHF3/O2 based gas	PT-OX
Residue clean	remove polyer residue using CF4 based gas	PT-OX
O2 plasma clean	O2 plasma etch 2 mins	Matrix
	**Sidewall Passivation**	
Conformal Al2O3 coating	deposit ~50 nm ALD Al2O3 500 cycles	Savannah
Conformal SiO2 coating	deposit ~250 nm PECVD SiO2	ccp
Oxide etch	etch ~250 nm oxide inside trench	PT-OX
Al2O3 etch	etch ~50 nm ALD Al2O3 inside trench	PT-MTL
	**Release etch**	
XeF2 etch	XeF2 etch 120~150 cycles	xactix
Parylene coating	deposit Parylene-C ~5 µm	PDS2010
Transfer and Assembly		
